# Assessment of novel antiviral filter using pseudo-type SARS-CoV-2 virus in fast air velocity vertical-type wind tunnel

**DOI:** 10.1038/s41598-023-41245-8

**Published:** 2023-08-25

**Authors:** Johnny Chun-Chau Sung, Pak-Long Wu, Ellis Yung-Mau So, Kam-Chau Wu, Sidney Man-Ngai Chan, Keith Wai-Yeung Kwong, Eric Tung-Po Sze

**Affiliations:** 1 Research Department, DreamTec Cytokines Limited, Hong Kong, China; 2 School of Science and Technology, Hong Kong Metropolitan University, Hong Kong, China

**Keywords:** Microbiology, Materials science, Techniques and instrumentation

## Abstract

Current evidence suggests that severe acute respiratory syndrome coronavirus 2 (SARS-CoV-2) can remain suspended spread in aerosols for longer period of time under poorly ventilated indoor setting. To minimize spreading, application of antiviral filter to capture infectious aerosols and to inactivate SARS-CoV-2 can be a promising solution. This study aimed to develop a method to assess simultaneously the filtration and removal efficiency of aerosolized pseudo-type SARS-CoV-2 using a vertical-type wind tunnel with relatively high face velocity (1.3 m/s). Comparing with the untreated spunlace non-woven filter, the C-POLAR™ treated filter increased the filtration efficiency from 74.2 ± 11.5% to 97.2 ± 1.7%, with the removal efficiency of 99.4 ± 0.051%. The results provided not only solid evidence to support the effectiveness of the cationic polymeric coated filter in fighting against the SARS-CoV-2 pandemic, but also a method to test viral filtration and removal efficiency under relative fast air velocity and with a safer environment to the operators.

## Introduction

As of May 2023, the outbreak of coronavirus disease 2019 (COVID-19) has caused more than 766 million cases and more than 6.9 million deaths globally^1^. The disease is caused by a positive-sense single-stranded RNA virus called severe acute respiratory syndrome coronavirus 2 (SARS-CoV-2)^2^ with spherical or elliptic morphology. The diameter of SARS-CoV-2 is approximately 60–140 nm, with a crown-like appearance due to the expression of spike glycoproteins on the envelope surface Some studies suggested that the spike glycoproteins are responsible for receptor binding and entering into the host cell^3,4^. It can transmit from human-to-human by multiple means, including short-range airborne transmission^5^ through atomization of SARS-CoV-2 in respiratory droplets (≥ 5 μm) and fine aerosols (< 5 μm) by inhalation, coughing or sneezing from an infected person^6^. Fears et al.^7^ also demonstrated that SARS-CoV-2 is persistent in aerosol suspension with the mass median aerodynamic diameter of around 2 μm.

In order to reduce the risk of infection, different types of filters have been thus deployed to reduce the aerosolized SARS-CoV-2, such as melt-blown polypropylene (MBPP) electret fabric^8^ and high-efficiency particulate air (HEPA) for heating, ventilation, and air conditioning (HVAC) system^9^. Since it is difficult to quantify virus in air samples, many of filtration efficiency studies were conducted by observing any residual viral RNA contamination on-site or using aerosol containing salt solution or bacteria as model to mimic viruses. There is a lack of standard method to directly assess the removal efficiency of virus^10^, especially for filters used in a HVAC or air purifier with relatively high face velocity, which remains uncertainty to the effectiveness of filter materials in prevention of SARS-CoV-2 infection.

Apart from reducing of bioaerosols through effective filtering, it is also important to inactivate the virus to prevent from fouling and secondary transmission. Some studies suggested the use of ultraviolet-C^11^ and dielectric filter discharge^12^ to inactivate SARS-CoV-2 bioaerosol, these systems have their own limitations including consumption of energy, and increase the ozone concentration in the treated air. Recently, a cationic polymeric coated filter system was introduced by C-POLAR™ Technologies, Inc. (https://cpolartechnologies.com), named as C-POLAR™ treated filter, consisting of a polyamine, a cationic polymer that are widely used as a gene delivery vector with high transfection efficiencies^13^. The C-POLAR™ material was used as coating on spunlace filter to increase the capture of negatively polar microbials in aerosol and to inactivate microbials through penetration of membrane and envelope by its high density of positively polar groups along the backbone chain.

**Figure 1:**
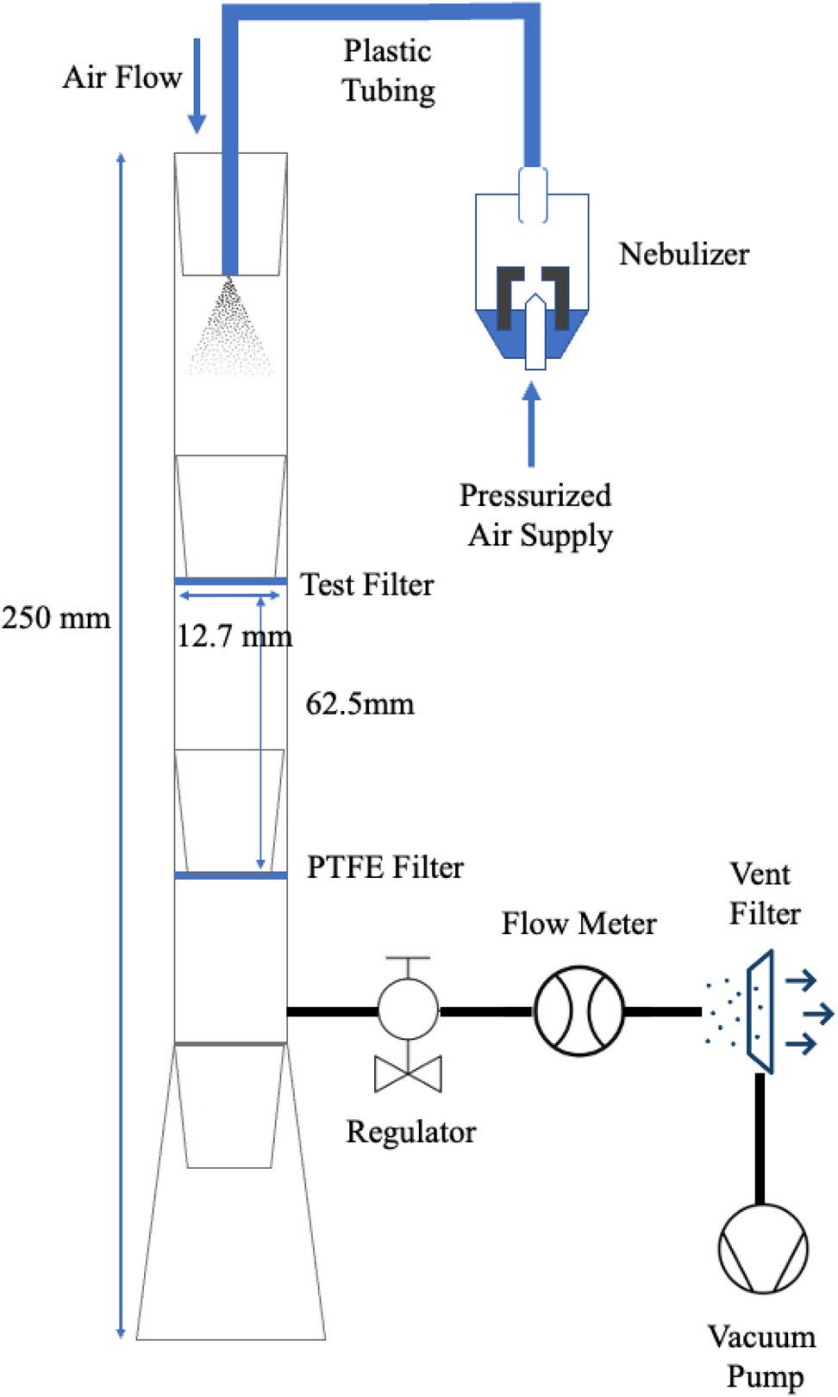
Schematic diagram of the vertical-type wind tunnel.

In this study, a vertical-type wind tunnel was developed (Fig. 1) to evaluate the filtration efficiency and anti-SARS-CoV-2 property of filter with C-POLAR™ treated filter. To assess the filtration efficiency with an airborne transmissible wild type virus, such as SARS-CoV-2, the health risk of generating aerosolized virus is much higher than culturing the same virus in a solution. The use of a safer alternative such as pseudo-type virus can reduce the health risk of operators and increase the accessibility to biosafety level 2 laboratories^14^. Pseudo-type virus is a genetical modified virus with attenuated virulence comparing with the wild-type, which get rid of the native surface protein expression and replaced with the desired surface proteins. The resulting pseudo-type virus can still infect the host cell but cannot replicate inside^15^. Importantly, the study provides a method to evaluate other filter system for filtration efficiency and antimicrobial properties in a relatively high air face velocity setting.

## Results and discussion

Compared with traditional assays, the use of pseudo-type virus assay demonstrated good correlation with wild-type SARS-CoV-2 assays, while maintaining a high throughput and requiring fewer biosafety requirements of the laboratory^16,17^. Although the use of virulence of the pseudo-type SARS-CoV-2 virus is much lower than the wild-type, the design of the wind tunnel was made under negative pressure using vacuum pump in order to minimize the risk of accidental leaks of pseudo-type virus to the atmosphere^18^, where the bioaerosols together with fresh air at the entrance would pass all the way through to the test (upstream), PTFE (downstream) and vent (HEPA) filter and release at the exhaust. The whole setup together with the exhaust gas were circulated inside the biosafety cabinet to ensure the operator’s safety during the experiment. The design of wind tunnel using a vertical configuration rather than a traditional horizonal type is to minimize the effect of aerosol and droplet particles settling onto the horizontal ducting surface by gravitational acceleration and increase the recovery of virus input^19^. The face velocity of the wind tunnel (i.e. 1.3 m/s) was comparable to the required by the ASHRAE Standard 52.2 for uniformity testing of the standard test duct^20^. The mass medium aerodynamic diameter of aerosol (i.e. 1.72 ± 0.26 μm) generated by the nebulizer was found similar to other studies for viral filtration performance^18,21,22^. McCluskey et al.^23^ specified aerosols within the size range of 0.5–20 μm are more likely to retain in the respiratory tract to cause the infection. Results from the study could be benchmarked with other testing methods to mimic bioaerosol removal efficiency in heating, ventilation, and air conditioning (HVAC) systems.

Table 1 summarized the infectious titer assay results (*n* = 3) of the virus input (directly collected at the entrance of plastic tubing from the nebulizer), and virus titers retained on the upstream and downstream filters. A consistent loading of pseudo-type virus in aerosol was delivered by the nebulizer to the wind tunnel, where a total of 9.60 × 10^5^ ± 5.17 × 10^4^ infectious units (IU) was aerosolized in each trial. The mean diameter of aerosol generated by the nebulizer, and the total amount of virus titers filtered by upstream and downstream filters in the control group (i.e. untreated spunlace non-woven filter) were found comparable to other wind tunnel studies^11,18^, where less virus volume as well as total virus titers were inputted in our experimental design. For the upstream filters, the average infectious units (IU) found on the cationic polymeric coated and untreated spunlace non-woven filters were found to contain 2.65 × 10^3^ ± 4.29 × 10^3^ IU and 4.47 × 10^5^ ± 4.03 × 10^4^ IU respectively. No detectable viable virus was found on 2 out of 3 C-POLAR™ treated filters under trial. The average and standard deviation viable counts for CPS filter were calculated with the use of detection limit (i.e. 1.03 × 10^2^ IU/filter).

**Table 1:** The infectious titer assay results of the virus input, and virus titers retained on the upstream and downstream filters.

Trial	Virus input/(IU)	Untreated spunlace non-woven filter (upsteam)/(IU)	PTFE filter (downstream)/(IU)	Filtration efficiency (%)	Catrionic polymeric coated filter (upstream)/(IU)	PTFE filter (downstream)/(IU)	Filtration efficiency (%)
#1	1.01E + 06	4.31E + 05	2.13E + 05	77.9	7.60E + 03	4.11E + 04	95.7
#2	9.07E + 05	4.92E + 05	1.58E + 05	83.5	N.D	3.17E + 04	96.7
#3	9.63E + 05	4.16E + 05	3.71E + 05	61.3	N.D	8.45E + 03	99.1
Average	9.60E + 05	4.47E + 05	2.47E + 05	74.2	2.65E + 03	2.71E + 04	97.2
Standard deviation	5.17E + 04	4.03E + 04	1.11E + 05	11.5	4.29E + 03	1.68E + 04	1.7

Filtration efficiency of samples was expressed in percentage. *ND* none detected.

**Figure 2:**
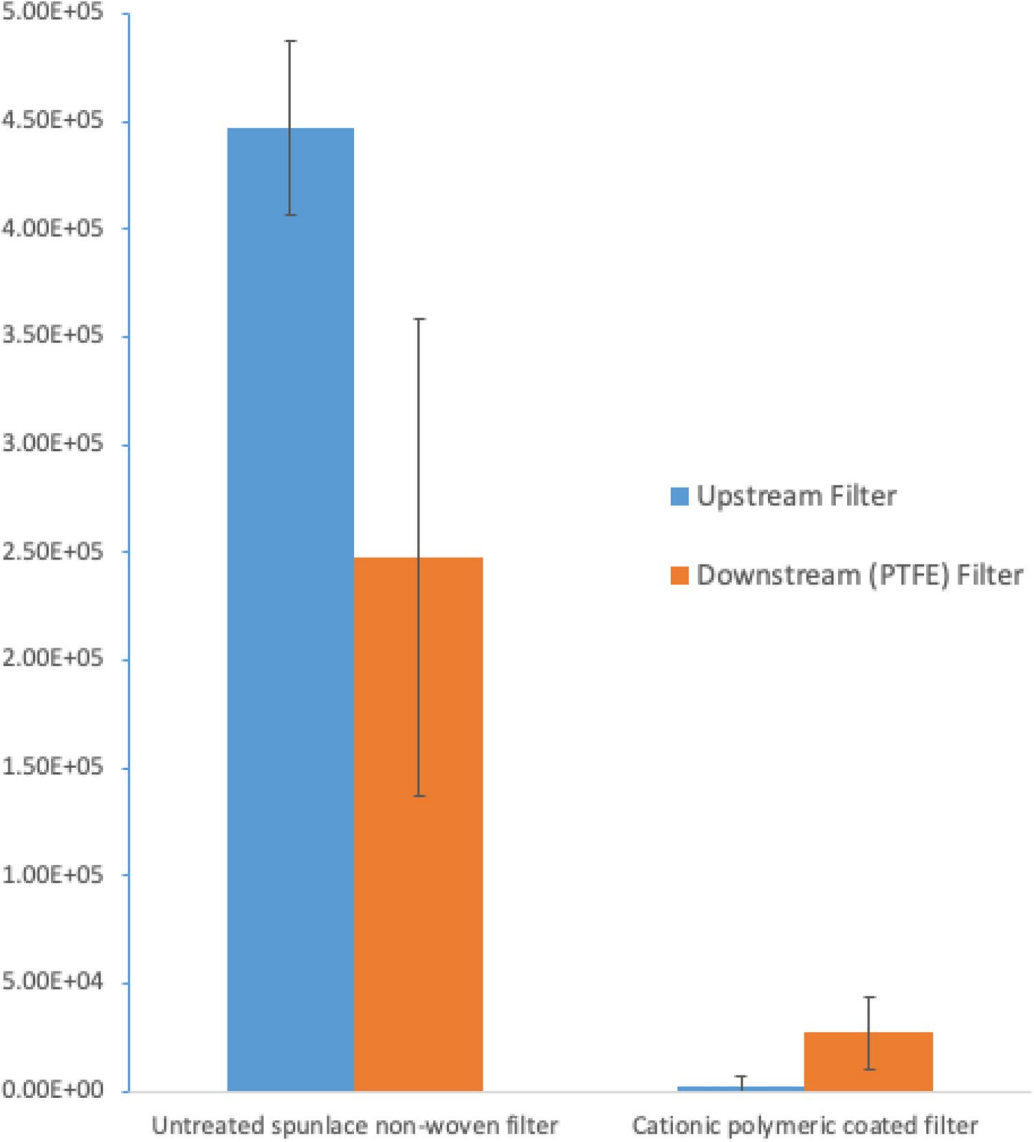
Results of virus titer retained on the upstream and downstream filters. Filtration efficiency of samples was expressed in percentage.

As shown in Fig. 2, there was a significant increase in filtration efficiency from 74.2 ± 11.5% for untreated spunlace non-woven filter to 97.2 ± 1.7% after the same filter coated with C-POLAR™ material (i.e. cationic polymeric coated filter) (2-sample *t *test, *p*-value = 0.036). There was also an order of magnitude decrease in titers found on downstream filter for the experiment of cationic polymeric coated filter, indicating a better trapping efficiency of the C-POLAR™ coated filter than untreated spunlace non-woven filter alone. The pH value of SARS-CoV-2 aerosol is generally alkaline and increased over time^24^, where the polarity of virus particles at neutral to alkaline pH would become negative as the virus’s isoelectric point is generally below 7^25^. The cationic polarization matrix induced on the filter surface by the polymeric C-POLAR™ material coated on the spunlace non-woven filter would thus enhance the capturing efficiency of virus particles in aerosol.

Compared with the results from Zhang et al.^21^ which used MS2 bacteriophage as the model, the viral filtration efficiency for the cationic polymeric coated filter was comparable to a minimum efficiency rating values (MERV) 14 filter under similar air face velocity. For the untreated spunlace non-woven filter, the filtration efficiency was slightly poorer than a MERV 12 filter, which indicated the application of positively polar C-POLAR™ material on the filter improving the strength in arresting bioaerosol and the resulting rating value.

By the use of infectious titer assay, the log reduction and removal efficiency of cationic polymeric coated filter was found to be 2.24 ± 0.038 and 99.4 ± 0.051% respectively, which imply the treated filter could inactivate pseudo-type SARS-CoV-2 effectively under relatively high face velocity. Compared with the use of dielectric filter discharge which inactivated SARS-CoV-2 by generation of ozone and other oxygen reactive species^12^, treated filter provide comparable removal efficiency of pseudo-type SARS-CoV-2 even at a much higher face velocity (1.3 m/s versus 0.18 m/s) and no energy consumption is required. Purwar et al.^22^ proposed a 3-layer omni-phobic coated polypropylene/copper coated diamond-like carbon (DLC) /non-woven (Hy-Cu) filter, which caused rupture to the lipid envelope of virus by the lipophobic property of the top layer and inactivate virus by copper surface on the middle layer. From its result of virus inactivation, the Hy–Cu filter with DLC was found to have 99% inactivation of MS2 bacteriophage virus over a period of 2 h (with the face velocity of 0.01 m/s), where the treated filter in this study can inactivate with more than 99% instantaneously just after the completion of aerosol spraying in 5 min.

The wind tunnel system in this study only assessed the viable virus by means of infectious titer assay coupling with quantitative polymerase chain reaction (qPCR) technique to determine the genome DNA from infected cells through transduction rather than the use of quantitative reverse transcription polymerase chain reaction (RT-qPCR) method to determine RNA recovered from filter extracts, as the RT-qPCR technique may create false positive results by detecting longer RNA fragments released from inactivated virus retained on the filters^26^.

## Conclusion

By the use of the vertical-type wind tunnel system developed, the ability of cationic polymeric coated filter in capturing of bioaerosols and inactivation of pseudo-type SARS-CoV-2 under were assessed experimentally. Bioaerosols passing through the cationic polymeric coated filter under tests showed a significant reduction by its 97.2 ± 1.7% filtration efficiency and 99.4 ± 0.051% removal efficiency, providing solid evidence to support the effectiveness of the cationic polymeric coated filter in fighting against the SARS-CoV-2 pandemic. The wind tunnel system can provide a consistent virus loading, relative fast air velocity and a safer environment to the operators. In the future, studies on the application flexible design vertical-type wind tunnel to filters will be conducted by the use of other microbial strains, and further improvement of the performance of the vertical-type wind tunnel system will be considered.

## Materials and method

### Preparation of C-POLAR™ treated filter

In this study, both the C-POLAR™ treated filter and untreated filter (i.e. polyester based non-woven spunlace filter) were obtained from C-POLAR™ technologies (Nevada, US), where the C-POLAR™ treated filter was prepared by applying a high-pressure stream of 6% C-POLAR™ polymer aqueous solution onto the untreated filter, followed by in-line drying the filter. Regarding the physical performance of the the C-POLAR™ treated filter, the minimum efficiency reporting values (MERV) in terms of particle filtration efficiency was increased from MERV 8 (for the untreated filter) to MERV 13 (for the C-POLAR™ treated filter), and the pressure drop of the C-POLAR™ treated filter was found to be around 31 pascals for the face velocity at 1.3 m/s. In this study, both the C-POLAR™ treated filter and untreated filter were cut into circular specimens with 25 mm diameter. Three samples for each filter were tested (i.e. *n* = 3).

### Pseudo-type SARS-CoV-2 virus

To reduce the safety risk of aerosolized virus, a pseudo-typed virus with the SARS-CoV-2 Spike protein expressed on the envelop surface carrying a green fluorescent protein (GFP) reporter gene was chosen as the test virus, which was produced in HEK293T cells according to the manufacturer’s instructions (InvivoGen, Hong Kong, China, cat. no. PLV-SPIKE)^27^. Briefly, 70–80% confluent HEK293T cells were transfected with envelope plasmid pLV-Spike, packaging plasmid and transfer plasmid carrying a GFP reporter gene with LentiTran transfection reagent (OriGene, Rockville, MD). 1-day post-transfection, the culture medium was removed, and the cells were washed with fresh medium twice to prevent plasmid DNA carryover. Cell media containing pseudo-typed viruses were then collected daily for 3 days. After collection, pseudo-typed virus containing media were centrifuged at 1000×*g* for 10 min and filtered through a 0.45-µm Polyethersulfone (PES) syringe filter (Thermo Fisher Scientific, Rockford, IL) to remove cell debris. The pseudo-typed viruses in the filtrate were then concentrated with Amicon® Ultra 15 mL Centrifugal Filters, 100 kDa nominal molecular weight limit (NMWL) (Merck Millipore, Darmstadt, Germany) before aliquoting and stored at − 80 °C before use.

### Virus filtration and inactivation test

The idea was to assess the filtration and inactivation properties of the novel coated filter to aerosol with pseudo-type SARS-CoV-2 virus under certain face velocity inside a wind tunnel. The setup and the experiments were conducted in a Class II biosafety cabinet (Esco Micro Pte. Ltd., Singapore), where temperature and humidity of the environment were maintained at 23 °C and 50% relative humidity respectively. The design of the vertical-type wind tunnel is illustrated in Fig. 1, where aerosol of pseudo-type SARS-CoV-2 virus in Dulbecco's Modified Eagle Medium (DMEM) was generated by a piston compressor type nebulizer (MEGANEB, Norditalia, Italy). The nebulizer was set to produce an aerosol with mass median aerodynamic diameter at 1.72 ± 0.26 μm (estimated by spraying with 5% potassium chloride solution as suggested by ASHRAE Standard 52.2 for experimental aerosol^20^) and a constant flow rate of around 0.2 ml/min for 5 min for each test. The total virus input from the nebulizer was measured by immersing the end of plastic tubing at the wind tunnel into a test tube with 5 mL of DMEM to collect the aerosol produced, followed by quantitation using infectious titer assay. For filtration efficiency and SARS-CoV-2 inactivation study, the aerosolized pseudo-type SARS-CoV-2 virus was diverted to the vertical-type wind tunnel with internal diameter 12.7 mm and length 250 mm, where the filter being tested and a polytetrafluoroethylene (PTFE) filter (0.22 μm pore size) were fitted at the upstream and downstream positions of the wind tunnel respectively to collect the aerosol with distance 62.5 mm apart. A vacuum pump was connected in orthogonal position after the downstream filter, where the flow rate was set at 10 L/min using an in-line type flow meter (LZB-10WB Senlod, Nanjing, China) to draw the aerosol and fresh air from the opening of the wind tunnel. The face velocity inside the wind tunnel was set at around 1.3 m/s. After 5 min of running aerosolized pseudo-type SARS-CoV-2 virus through the filters in the above-mentioned conditions, both the nebulizer and the vacuum pump were switched off and both the upstream and downstream filters were extracted separately in 10 mL DMEM using vortex. Viable virus retained on the filter that extracted by DMEM was quantified by infectious titer assay. The filtration efficiency of the filters being tested was determined by the equation below, with the use of the virus titers retained on the downstream filter and the virus input:

1 $$Filtration \,\,Efficiencey=\frac{Virus\,\, input-virus \,\,on\,\, downstream\,\, filter }{Virus\,\, input}\times 100\%.$$

For the viral inactivation, the log reduction and removal efficiency of the C-POLAR™ treated filter was determined by comparing the titers found in the infectious titer assay between the untreated and C-POLAR™ treated filters^11^:

2 $$Log\,\, reduction={\mathrm{log}}_{10}\left(\frac{average\,\, titers \,\,on\,\, uncoated\,\, filter}{average\,\, titers \,\,on\,\, C-POLAR^{TM}\, treated\,\, filter}\right),$$

3 $$Removal\,\, efficiency =\left(1-{10}^{-Log\,\, reduction}\right)\times 100\%.$$

### Infectious titer assay

To quantify pseudo-type SARS-CoV-2 virus retained on each of the tested filter, adherent human ACE2 and TMPRSS2 expressing A549 cells (InvivoGen, Hong Kong, China, cat. no. a549-hace2tpsa) cells were incubated with the serially diluted DMEM extract from the tested filter. Cells infected by pseudo-type SARS-CoV-2 virus in the diluted extract was measured by quantitative polymerase chain reaction (qPCR) as described by Barczak et al.^28^ with some modifications. Briefly, 1 × 10^4^ A549 adenocarcinomic human alveolar basal epithelial cells with the expression of human angiotensin converting enzyme 2 (ACE2) and transmembrane serine protease 2 (TMPRSS2) were cultured in a 96-well plate at 37 °C with 5% CO_2_ in a humidified incubator (Thermo Fisher Scientific, Rockford, IL). DMEM supplied with 10% Fetal Bovine Serum (FBS) and 1% penicillin/streptomycin (Thermo Fisher Scientific, Rockford, IL) were used as cell culture media. Infections of pseudovirus were performed by adding diluted filter extract to the culture media for cell culturing. Culture medium without addition of filter extract was used as negative control. Viable pseudo-type SARS-CoV-2 virus remained in the filter extract can infect the A549 cells and integrate its genomic sequence into the A549 cell’s genome through transduction. Upon 3-day post-infection, the genomic DNA were extracted from the cultured cells using Monarch® Genomic DNA Purification Kit (New England Biolabs, Ipswich, MA) according to manufacturer’s instruction. To measure the infectious titer, qPCR was performed by targeting the lentiviral-specific transgene (i.e. Woodchuck Hepatitis Virus Posttranscriptional Regulatory Element (WPRE)) and a single copy refence gene (i.e. albumin (ALB)). qPCRs were performed in a QuantStudio™ 3 Real-Time PCR System (Thermo Fisher Scientific, Rockford, IL) in triplicates with the reaction mixture containing 10 μl Luna® Universal qPCR Master Mix (New England Biolabs, Ipswich, MA), 800 nM for each primer (forward and reverse), 5 μl of the genomic DNA and make up the final volume to 20 μl with nuclease-free water. The thermocycling programme to amplify the lentiviral-specific fragment (WPRE) and albumin gene (Alb) was as follows: 95 °C for 5 min (initial denaturation and polymerase activation), followed by 40 cycles at 95 °C for 15 s, 60 °C for 30 s, and 72 °C for 10 s (detection). A melting curve analysis (60–95 °C range) was conducted at the end of the reaction to verify specificity of the qPCR product, where the melting temperature of the WPRE gene and albumin were at Tm = 84.5 °C and Tm = 77.2 °C respectively. In each run of qPCR, ten-fold serial dilutions of the transfer plasmid for the pseudo-typed virus preparation in known concentration were prepared to create calibration curve for quantification. A no template sample was used as negative control.

Primers used for the qPCR:

ALB forward 5′-TTTGCAGATGTCAGTGAAAGAGA-3′.

ALB reverse 5′-TGGGGAGGCTATAGAAAATAAGG-3′.

WPRE forward 5′-GTCCTTTCCATGGCTGCTC-3′.

WPRE reverse 5′-CCGAAGGGACGTAGCAGA-3′.

### Statistical analysis

Data are expressed as the mean ± standard error of the mean (SEM), and statistical significance was determined by student’s *t* test in GraphPad Prism 7.1 (San Diego, CA, USA). Data were considered significantly difference when the *p*-value was less than 0.05.

## Data Availability

The data that support the findings of this study are available on request from the corresponding author, E.T.-P.S.
